# Internal short circuit detection in Li-ion batteries using supervised machine learning

**DOI:** 10.1038/s41598-020-58021-7

**Published:** 2020-01-28

**Authors:** Arunava Naha, Ashish Khandelwal, Samarth Agarwal, Piyush Tagade, Krishnan S. Hariharan, Anshul Kaushik, Ankit Yadu, Subramanya Mayya Kolake, Seongho Han, Bookeun Oh

**Affiliations:** 1 Mobile Battery Research Lab, Samsung R&D Institute India - Bangalore (SRIB), #2870, Phoenix Building, Bagmane Constellation Business Park, Outer ring road, Doddanekundi circle, Marathahalli Post, Bangalore, 560037 India; 2 Advanced Battery Lab, Mobile Communication Business Division, SAMSUNG ELECTRONICS Co., Gyeonggi-do, 16677 Korea

**Keywords:** Chemical engineering, Computational science

## Abstract

With the proliferation of Li-ion batteries in smart phones, safety is the main concern and an on-line detection of battery faults is much wanting. Internal short circuit is a very critical issue that is often ascribed to be a cause of many accidents involving Li-ion batteries. A novel method that can detect the Internal short circuit in real time based on an advanced machine leaning approach, is proposed. Based on an equivalent electric circuit model, a set of features encompassing the physics of Li-ion cell with short circuit fault are identified and extracted from each charge-discharge cycle. The training feature set is generated with and without an external short-circuit resistance across the battery terminals. To emulate a real user scenario, internal short is induced by mechanical abuse. The testing feature set is generated from the battery charge-discharge data before and after the abuse. A random forest classifier is trained with the training feature set. The fault detection accuracy for the testing dataset is found to be more than 97%. The proposed algorithm does not interfere with the normal usage of the device, and the trained model can be implemented in any device for online fault detection.

## Introduction

**Figure 1:**
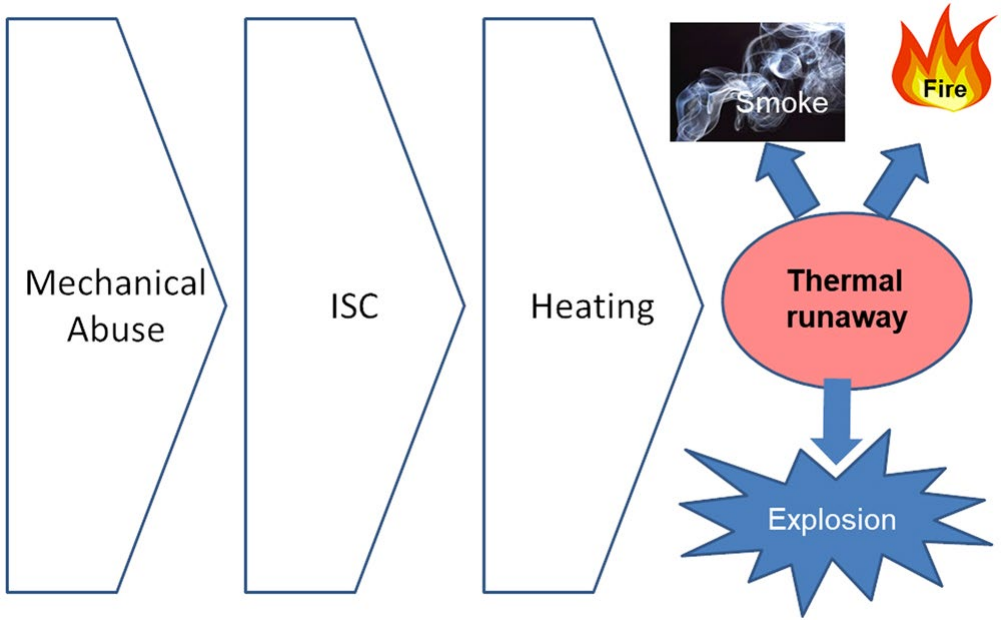
Mechanical abuse to thermal runaway mechanism.

Nowadays, smart phones, electric vehicles and most of consumer electronics use Li-ion batteries (LiBs) due to their high energy density, long cycle life and extended calendar life. Due to the wide spread applicability, LiBs are subjected to mechanical abuse of varying intensities. As illustrated in Fig. 1, mechanical abuse to the battery may lead to internal short circuit (ISC) due to the damage of the insulating separator, deflection of the electrodes, etc.^1^. Such ISC causes internal heating and further damage of the battery, that may cause smoke, fire or an explosion. Thermal runaway of the battery is a serious threat to user safety.

The effects of the mechanical abuse on the LiB and the mechanisms of thermal runaway have been studied extensively in the literature by modelling and experiments. The models have been developed by combining the mechanical, electrochemical, and thermal behaviour of the LiBs under various types of mechanical abuses^2–9^. Most of the reported models have been validated with experimental data. The dynamic and quasi-static mechanical abuse tests studied by several researchers are indentation test^4,10,11^, punch test^12^, nail penetration test^8^, pinch-torsion test^13^, compressive test^14–16^, drop test or impact test^17^, etc. The mechanism of ISC development from the pinch and pinch-torsion types of mechanical abuse has been modelled in^18^ and stated that pinch-torsion is more effective than the pure pinch in puncturing the separator and creating ISC. Fracturing of the separator due to the ground impact of an electric vehicle battery which leads to ISC formation has been modelled using global finite element^19^. The crush test has been performed^20^ on the whole battery pack of four cells and the short circuit current has been measured. The short circuit resistance has been estimated from the measured current. A mechanical model of cylindrical LiB has been developed^21^ for the vehicle crash simulation. Impact test has been performed using a drop tower on fully charged batteries^17^. The effect of loading and the impact location on the probability of short circuit and thermal run away have been studied in detail. A detailed review of the existing experimental and model based methods to study the effect of mechanical abuse on the different form factors of Li-ion batteries is available^22^.

The stress-strain distribution under abusive conditions and the relationship with the ISC formation have been reported^4,23^. The mechanical properties and the failure mechanism of the different battery components such as the electrodes^24–26^ and the separators^24,27^ have also been studied individually. The mechanical properties of the overall battery pack has been explored^28^. The effect of design parameters such as the porosity and thickness of the electrodes and separators on the ISC caused by mechanical abuse has been investigated^6^. Researchers have reported two different failure modes of the separators^12^, and concluded that one of them causes thermal runaway and the other one may not exhibit such severe effect. Experiments have been performed^29^ using dummy pouch cells without the conductive salts to determine the short circuit resistance value under different compressive loads. Few possible ways to prevent or reduce the effect of the thermal runaway followed by the mechanical abuse have been discussed^9^. The effect of state of charge (SOC) levels on the consequence of the mechanical abuse has been researched^23^ using simulation and experimentation. The researchers also suggested few thermal run away detection strategies based on force-displacement, voltage-temperature^30^, stress-strain, SOC^31^, over-discharge^32^ etc. A quantitative definition of the state of safety (SOS) has been provided as the inverse of safety^33^.

The aforementioned literature on the mechanical abuse of LiBs leading to ISC formation and the subsequent thermal runway is quite insightful. It helps to improve the battery safety design and the assessment of hazard under the real world mechanical abuses. However, the detection of ISC followed by the mechanical abuse is also an important issue for the battery and user safety. Many effective methods have been reported in the literature for ISC detection using a range of statistical measures, estimation techniques, observer designs, etc. The correlations between the different voltage curves of various cells present in a battery pack have been used to detect the short circuits^34^. The external short circuit has been identified in^35^ using the Gaussian classifier on the features extracted by maximum likelihood estimator from the battery current and voltage data. Different observers, such as extended Kalman filter (EKF)^36^, Luenberger observer^37^, etc. have been employed to generate the residual signal from the measured data for the ISC detection. In another approach, the researchers have estimated various informative parameters from the measured battery current, voltage, and temperature data and compared them with their normal values for the ISC detection in^38–41^. There are few patents covering the topic of LiB ISC detection. The methodologies reported in the patents check for the battery temperature level^42^, voltage values at different SOC levels^43^, instantaneous change in battery voltage^44^ and current^45^, battery resting voltage change over a long period of time^46,47^, etc.

Amongst all the batteries undergoing mechanical abuse, not all necessarily result in to ISC. We have also observed the same from our several experiments of dropping the batteries. It is also observed that some features revert to healthy regime upon cycling and some appear in faulty regime. This may be due to the non-uniform deformation of electrode layers of jelly rolled pouch cell. The real life ISC due to non-rigorous quality control practiced during manufacturing process is akin to the feature response shown by the dropped battery. Hence, classification of healthy and faulty battery using simple statistical classification based on single feature does not yield good accuracy. Therefore, in this work we have developed a robust supervised learning method for robust classification of healthy and faulty batteries using random forest (RF) based classifier. RF uses an ensemble of trees of slightly different structures for classifications. The use of multiple trees makes RF more robust compared to any single classifier^48^ and the accuracy is also high^49^. Compared to other classifiers like Artificial Neural Networks (ANN), nearest neighbour, Support Vector Machine (SVM), RF is a linear classifier and showed better results in our analysis. Further, scaling of the features is not an issue. The computational complexity of RF is low compare to few other popular classifiers^50^ and it serves as a lightweight online algorithm that does not interfere with normal usage. RF has been applied successfully to various classification problems such as sleep stages classifications from the electroencephalography (EEG) data^51^, bearing faults identification from the vibration data^52^, facial expression detection from video data^53^, crops type classification from the hyper spectral images^54^, lung vessel segmentation^55^ from computed tomography (CT) image, etc.

The literature is rich with the methods of ISC detection in the LiBs. However, the following reasons limit the applicability of the available methods for the detection of mechanical abused induced ISC in the smart phone’s LiB.

1. Some of the reported methods are only applicable to the battery packs where multiple cells of similar specification are present^34,56^.
2. In the smart phones, the battery temperature^40,42,57^ at the appropriate spot may not be available. In addition, the temperature rise happens just before the thermal runaway, therefore early warning may not be possible.
3. In case of the smart phones, the normal device operation cannot be hampered by any special charge-discharge profiles^46,47,58,59^.

In this paper, an algorithm has been developed for online detection of the mechanical abuse induced ISC in the smart phone’s LiB using the previously mentioned RF classifier. We have also built an Android application to log the battery charge-discharge data inside the smart phones. The recorded data then transferred to the computer for further processing. First, a set of features have been extracted from the training dataset of battery current and voltage for classification purpose. The training dataset is composed of healthy and ISC battery data. The ISC condition has been emulated by connecting external short circuit resistances across the battery terminal, and the battery has been connected to the phone for regular charge-discharge. The RF classifier has been trained using the features extracted from the training dataset. The testing dataset comprises of healthy and abused (with ISC) battery data. The LiBs have been abused by dropping them on hard surface. This allows the test conditions to be random, which simulates real life situations. The abused batteries with ISC and the healthy batteries have been classified with the trained RF model. In the proposed methodology, the training will be performed offline using the healthy and ISC (emulated by external short circuit resistances) battery data, and then the trained classifier will be ported in the mobile device for online detection of ISC induced by the mechanical abuses or any other reasons.

## Results and Discussion

**Figure 2:**
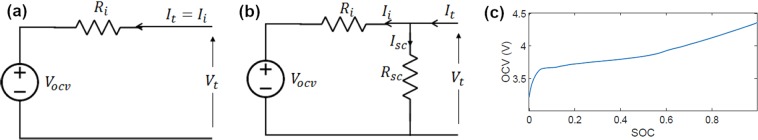
Battery electrical equivalent circuit for (**a**) Healthy battery, (**b**) Faulty battery and (**c**) SOC vs OCV curve during discharge.

The following equivalent circuit models (ECM)^60^ are used for healthy (Fig. 2a) and faulty (ISC) (Fig. 2b) LiBs.

*V*_*t*_ and *I*_*t*_ are the terminal voltage and current respectively. *V*_*ocv*_ is the open circuit voltage (OCV) of the battery. *R*_*i*_ is the internal resistance of the battery which is a function of SOC. *R*_*sc*_ is the short circuit resistance and *I*_*sc*_ is the leakage current or the short circuit current. The relationship of OCV with the terminal voltage and current for healthy 1 and ISC 2 cases are as follows

1 $${V}_{t}(k)={V}_{ocv}(k)+{R}_{i}(SOC){I}_{t}(k)$$

2 $${V}_{t}(k)={V}_{ocv}(k)+{R}_{i}(SOC){I}_{i}(k)$$

where under healthy case, *I*_*i*_(*k*) = *I*_*t*_(*k*), and ISC case, *I*_*i*_(*k*) = *I*_*t*_(*k*) − *I*_*sc*_(*k*) and ${I}_{sc}=\frac{{V}_{t}(k)}{{R}_{sc}}$.

A set of features are used to encompass the effect of ISC on the battery voltage and current. In the subsequent subsections, the features and the reason for individual choices are discussed.

### End of charge SOC (*SOC*_*max*_)

The SOC is estimated as follows:

3 $$SOC(k+1)=SOC(k)+\frac{{I}_{t}(k){T}_{s}}{{C}_{max}}$$

where *T*_*s*_ is the sampling time, and *C*_*max*_ is the maximum capacity of the battery.

In the case of ISC, SOC at the end of charging will be more than the normal case because of the leakage current (*I*_*sc*_).

### OCV at low SOC level (*V*_*cmin*_)

The OCV (*V*_*cmin*_) 4 at *p*% user SOC during the discharging phase is taken as a feature. In the case of ISC fault, the actual SOC will be lower than the estimated user SOC from the terminal current because of the presence of the leakage current *I*_*sc*_. The difference will increase with time as the actual SOC reduces. Since OCV is a monotonic function of SOC, *V*_*cminf*_ will be lower than *V*_*cminh*_, where the subscripts *f* and *h* denote faulty and healthy cases respectively.

4 $${V}_{cmin}={V}_{t}(at\,p \% \,user\,SOC)+{R}_{i}(at\,p \% \,user\,SOC){I}_{t}(at\,p \% \,user\,SOC)$$

### CV time (*T*_*cv*_)

Typically, battery charging is performed using the protocol of constant current (CC) or constant power (CP) charging followed by constant voltage (CV) charging^61,62^. The time taken to complete the constant voltage (CV) phase (*T*_*cv*_) of charging is also a useful feature for ISC detection. Since a portion of the charging current always flows through the short circuit path, the faulty battery takes more time to complete the CV phase, *T*_*cvf*_ > *T*_*cvh*_. The CC or CP charging time also increases under ISC fault. However, the percentage of charging current flowing through the short circuit path during CC or CP charging is much smaller compared to the CV phase. The difference between the charging time for the healthy and faulty cases is more in the CV phase then in the CC phase. Therefore, the effect of ISC is more prominent in the CV phase.

### Energy loss (*E*_*L*_)

In the case of ISC fault, the total energy input to the battery during the charging phase will be more and the total energy output during the discharging phase will be less compared to the healthy case. The continuous loss of energy through the short circuit resistance $\left(\Sigma \frac{{V}^{2}(k)}{{R}_{sc}}{T}_{s}\right)$ causes this difference. Therefore, the difference of energy between the charge and discharge phase is taken as a feature 5, *E*_*Lf*_ > *E*_*Lh*_.

5 $${E}_{L}=\sum _{charging}{V}_{t}(k){I}_{t}(k){T}_{s}-\sum _{discharging}{V}_{t}(k){I}_{t}(k){T}_{s}$$

### Slope of OCV near the end of discharge (*Slope*_*ocv*_)

The *Slope*_*ocv*_ feature is evaluated as

6 $$Slop{e}_{ocv}=\frac{{V}_{ocv}(m)-{V}_{ocv}(n)}{SOC(m)-SOC(n)}$$

where the *m*-point correspond to the end of discharge and the *n*-point is identified in such a way that, *SOC*(*m*) − *SOC*(*n*) = 5%. As discussed earlier, the actual SOC remains lower than the estimated SOC under ISC fault. The lower SOC during ISC pushes the operating point towards the high slope region of the SOC-OCV curve (Fig. 2c). Therefore, *Slope*_*ocvf*_ > *Slope*_*ocvh*_.

### *SOC* vs. *V*_*ocv*_ slope (*a*_1_)

The OCV will drop faster with respect to the estimated SOC during the discharge because of the continuous leakage under ISC fault. Therefore, a fifth order polynomial is fitted 7 between *V*_*ocv*_ and estimated *SOC*. The first order slope parameter *a*_1_ is taken as a feature. A fifth order polynomial was found to give a good fit with data. Higher order polynomials gave an ill-conditioned system to solve for the polynomial co-efficients.

7 $${V}_{ocv}(k)={a}_{0}+{a}_{1}.SOC(k)+{a}_{2}.SO{C}^{2}(k)+{a}_{3}.SO{C}^{3}(k)+{a}_{4}.SO{C}^{4}(k)+{a}_{5}.SO{C}^{5}(k)$$

### Internal resistance

Battery internal resistance (*R*_*i*_) is an important parameter to detect ISC. *R*_*i*_ is evaluated for the existing implementation as,

8 $${R}_{i}(SOC(k))=\frac{\left|{V}_{t}(k)-{V}_{ocv}(k)\right|}{{I}_{t}(k)}.$$

*V*_*ocv*_ values are gathered up from the SOC-OCV relationship. The estimated *R*_*i*_ under normal 9 and ISC 10 conditions during the charging phase will be as follows. The subscript *c* is used to indicate charging phase. It is clear from 9 and 10 that ${\widehat{R}}_{ifc} < {\widehat{R}}_{ihc}$,

9 $${\widehat{R}}_{ihc}(SOC(k))=\frac{{V}_{t}(k)-{V}_{ocv}(k)}{{I}_{t}(k)}={R}_{i}(SOC(k))$$

10 $${\widehat{R}}_{ifc}(SOC(k))=\frac{{V}_{t}(k)-{V}_{ocv}(k)}{{I}_{t}(k)}={R}_{i}(SOC(k))-\frac{{R}_{i}(SOC(k))}{{R}_{sc}}\frac{{V}_{t}(k)}{{I}_{t}(k)}$$

The estimated *R*_*i*_ under normal 11 and ISC 12 conditions during the discharging phase will be as follows. The subscript *d* is used to indicate discharging phase. It is clear from 11 and 12 that ${\widehat{R}}_{ifd} > {\widehat{R}}_{ihd}$.

11 $${\widehat{R}}_{ihd}(SOC(k))=\frac{{V}_{ocv}(k)-{V}_{t}(k)}{{I}_{t}(k)}={R}_{i}(SOC(k))$$

12 $${\widehat{R}}_{ifd}(SOC(k))=\frac{{V}_{ocv}(k)-{V}_{t}(k)}{{I}_{t}(k)}={R}_{i}(SOC(k))+\frac{{R}_{i}(SOC(k))}{{R}_{sc}}\frac{{V}_{t}(k)}{{I}_{t}(k)}$$

Under the ISC fault, the estimated resistance decreases during charging and increases during discharging compared to the healthy case. Hence the difference between the estimated charging and discharging resistances will reduce in the case of the ISC fault, i.e. (\10–12∣) will reduce compared to (∣9–11∣). Another observation can be made from 10 and 12, that under the ISC fault, the change in the estimated ${\widehat{R}}_{i}$ will be more during the discharging phase compared to the charging phase. The effect of ISC is prominent in the discharging phase estimated resistance because the magnitude of current remains smaller compared to the CC or CP charging current for smart phone applications. From the two observations, the following two features are extracted from the estimated internal resistance.

1. *ΔR* =  (estimated resistance at the end of charging)  −  (estimated resistance at the start of discharging)
2. *meanR* =  mean of the estimated resistance for the window of SOC 0.3 to 0.8 during discharging

It must be pointed out that using various methods in literature for the direct estimation of *R*_*sc*_ a very high variance in estimates was found and an alternative was needed. Instead the estimation of ${\widehat{R}}_{i}$ was done using a Kalman Filter and did not require the estimation of *R*_*sc*_. A process noise with zero mean and covariance = 10^−3^ and an observation noise with zero mean and covariance = 25 has been assumed.

**Figure 3:**
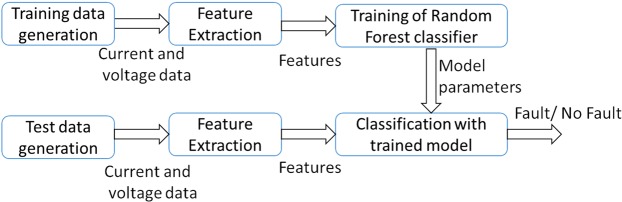
Block diagram of the proposed methodology.

The block diagram of the proposed methodology is shown in Fig. 3. The method is explained in a systematic way in the following steps.

1. Data generation for training and testing purpose.
2. A set of 8 features as are extracted from the battery current and voltage data during standard charging and discharging.
3. Random Forest (RF) classifier is trained with the training set features.
4. Testing set features are used to test the performance of the trained classifier for the identification of healthy and abused batteries with ISC.

For the training dataset, optimum values need to be found for the number of trees (*N*_*t*_) and the number of features (*max*_*features*) in the RF problem. As explained further in the Methods section, a grid search is performed over the feasible range of *N*_*t*_ and *max*_*features* to find their suitable values for the training dataset. Separate grid searches are performed for different end of discharge SOC levels and the derived *N*_*t*_ and *max*_*features* values change accordingly. For example, when the end of discharge SOC level is 5%, the values of *N*_*t*_ = 15 and $max\,\_features=\sqrt{total\_number\_of\_\,features}$ are derived.

**Figure 4:**
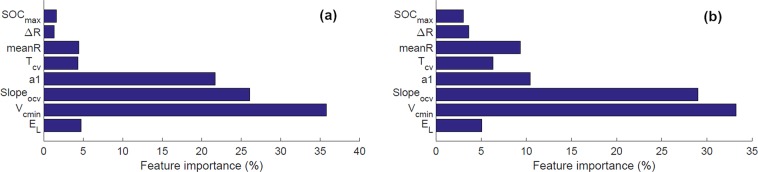
Feature importance matrix for different SOC cutoff limits, (**a**) SOC cutoff 5%, (**b**) SOC cutoff 15%.

The importance of different features for the healthy and faulty classifications is shown in Fig. 4. The results are generated using the training dataset (healthy and external short cases) for different end of discharge SOC. The importance of features changes depending on the end of discharge SOC value. After analysing the sub-figures in Fig. 4, we can conclude that *V*_*cmin*_, *Slope*_*ocv*_, *a*_1_, *meanR*, and *E*_*L*_ are the most important features in classifying the healthy and faulty classes from the training dataset. However, the other features also contribute to certain extents. It has been verified that accuracy falls down if the number of features is reduced. The Gini index is used to find the quality of a split in the Python package scikit-learn. The mean decrease in impurity approach is applied to the fraction of samples a feature contributes, to create a normalized estimate of the importance of that feature. In generating these features a fixed value of ${\widehat{R}}_{i}=0.1\Omega$ has been used.

The RF classifier has been trained using the healthy and ISC battery (with external short circuit resistance) data for different end of discharge SOC levels. The parameters derived from the grid search are used for the final training. Users may not always discharge the battery to a very low SOC value under the practical cases. Therefore, one of the trained classifiers can be used to predict the battery health from the test data depending on the percentage of partial discharge. The performance of the classifier for the training data for a SOC cut-off of 5% is found to be Normally Normal (NiN) = 100%, Faulty in Fault (FiF) = 99.66%, False Alarm = 0.0% and a Miss detection = 0.34%. The training performance is not affected much by different SOC cut-offs. Total 290 faulty cycles of different magnitudes of external short (150 Ω to 500 Ω) and 53 healthy cycles are used to train the RF classifier and generate the confusion matrices.

**Figure 5:**
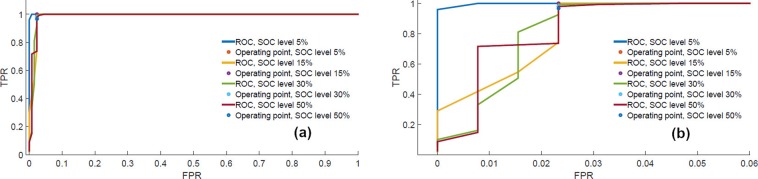
ROC curves for testing data for different end of discharge SOC levels (**a**) Full view (**b**) Expanded view.

The trained RF models are used to classify the abused batteries with ISC from the healthy population. 129 faulty (ISC induced by mechanical abuse) and 148 healthy charge-discharge cycles from 5 different batteries are used for testing. Testing is also performed for different end of discharge SOC levels similar to the training dataset. The receiver operating characteristics (ROC) curves for different level of partial discharging (end of discharge SOC) are shown in Fig. 5. All ROCs have area under the curve(AUC) close to 1 which means the trained RF models can also classify the abused batteries with ISC faults with high accuracy. The performance of the classifiers degrade minutely with the increase of end of discharge SOC levels. Confusion matrix represents the performance of a classifier in the tabular form. Rows of the matrix specify the actual class and columns specify the predicted class. For the two-class classification problem, the first, second, third and fourth quadrants of the 2 × 2 matrix represent false positive (FP), true negative (TN), false negative (FN) and true positive (TP) numbers respectively. The confusion matrices for the operating points marked on the ROC curves are shown in Table 1. The confusion matrices also infer that the proposed method has good detection accuracy with low miss-detection and false alarm rates for different end of discharge SOC levels.

**Table 1:** Confusion matrix for testing dataset for different SOC cut-off limit.

			NiN = 100%				NiN = 97.97%
**SOC = 5**%	Faulty	Healthy	FiF = 98.45%	**SOC = 15**%	Faulty	Healthy	FiF = 97.67%
Faulty	TN = 127	FP = 2	False Alarm = 0.0%	Faulty	TN = 126	FP = 3	False Alarm = 2.03%
Healthy	FN = 0	TP = 148	Miss-detection = 1.55	Healthy	FN = 3	TP = 145	Miss-detection = 2.33%
			NiN = 97.97%				NiN = 93.92%
**SOC = 30**%	Faulty	Healthy	FiF = 97.67%	**SOC = 50**%	Faulty	Healthy	FiF = 97.67%
Faulty	TN = 126	FP = 3	False Alarm = 2.03%	Faulty	TN = 126	FP = 3	False Alarm = 6.08%
Healthy	FN = 3	TP = 145	Miss-detection = 2.33	Healthy	FN = 9	TP = 139	Miss-detection = 2.33%

The performance of RF was compared with different classifier and the results are shown in Table 2. The same data was used to train and test all classifiers. It can be seen that the value for NiN is the highest for RF.

**Table 2:** Performance of different classifiers.

Classifier	RF	ANN	SVM
NiN	100.0	97.97	94.59
FiF	98.45	100.0	87.60

## Conclusion

A novel methodology with high accuracy is proposed for online detection of mechanical abused induced ISCs in the smart phone batteries. The proposed methodology is tested successfully with 100% normal in normal and 98.45% fault in fault accuracy for the complete discharge cases (end of discharge SOC = 5%). Advanced supervised machine learning is applied to learn about the healthy and faulty feature spaces from the healthy and pure short data. The trained models are then applied to detect the mechanical abuse induced ISC faults in the smart phone batteries. The batteries are abused by dropping them from 4ft height on the hard surface. Three batteries are used for generating training data and five batteries are used for testing purpose. Training and testing subjects are completely different from each other. The proposed algorithm has been tested in a computer using the data logged in the smart phones using the developed Android APK. In the proposed methodology, the training of the classifier can be performed in the same way using a PC, and then the trained model will be transferred to the smart phones in the form of APK or as embedded code for the power management integrated circuit (PMIC) for online detection of ISC. The proposed methodology can also be applied after certain modifications to detect ISCs in the Li-ion batteries used in the various systems such as EVs, aircrafts, etc. induced by mechanical abuses or other factors. The benefits of the methodology are that it can be used to detect a pre-existing fault and detect ISC from the first cycle itself. There are no restrictions on the SOC range of cycling of the battery and the algorithm is capable of detecting ISC for any partial charging cycle. However a continuous range of charge or discharge data is required for the algorithm to work. This limitation is planned to be addressed in future studies. Finally, given that 8 different features are combined to predict faults makes the methodology more robust and reliable. All this is done keeping in mind that the methodology is simple enough and does not add a significant overhead to device operation.

## Methods

### Training and Testing data generation

An Android application has been developed to log the battery current and voltage from the PMIC while charging and discharging the smart phone. Li-ion rechargeable smartphone batteries with 3 Ah capacity and 3.85V nominal voltage have been used. The charging scheme used is the normal constant current (CC) charging at 0.7*C* rate followed by the constant voltage (CV) charging. The display and the flashlight of the phone are kept on and the processor is loaded with some dummy programs to discharge the battery.

**Figure 6:**
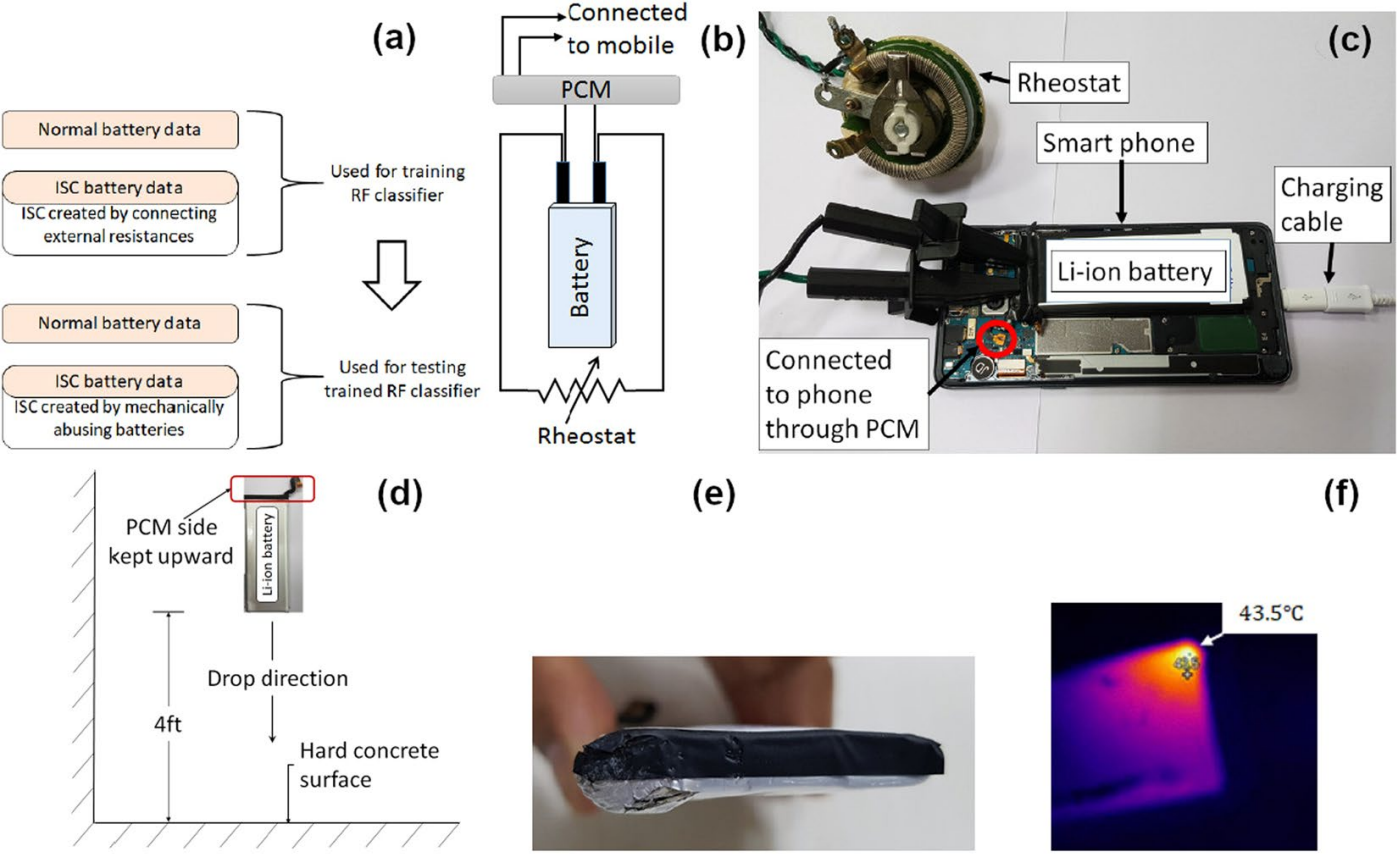
Training and testing battery data generation arrangements. (**a**) Types of data and usages. (**b**) Schematic diagram of the ISC test setup. (**c**) Experimental setup of ISC test (**d**) Schematic diagram of the drop test (**e**) Dropped battery (**f**) Thermal image of dropped battery.

The Fig. 6a illustrates the different types of battery data generated for this research and their utilization. The train data set contains both the normal and the ISC battery (with external resistance) data from three different batteries for several charge-discharge cycles. External short circuit resistances of various values are connected across the battery terminals when the battery is also connected to the phone to generate the ISC data. The short circuit resistance value is changed in steps from 500 Ω to 150 Ω. The schematics of the electrical circuit of such arrangements is shown in Fig. 6b, and the photograph of the setup is shown in Fig. 6c.

Testing is performed on the five different healthy and subsequently abused batteries to detect ISCs using the trained RF model. First, the healthy batteries are cycled in the devices using the developed application and the healthy data are logged. Then those batteries are abused by dropping them from 4*ft* height on the hard surface with the protection circuit module (PCM) kept upside. A schematic diagram of the battery dropping arrangements is shown in Fig. 6d. Figure 6e shows a dropped battery with deformed corner due to the impact. Figure 6f shows the thermal image of the dropped battery with the hotspot generated at the deformed location. The most likely mechanism of short here seems to be the physical deformation of the battery which leads to a conductive path between the electrodes. The abused batteries are then cycled in the devices and the testing data are logged. Batteries developing ISC due to drop is a probabilistic event. The batteries may not develop ISC even after the drop. Therefore, after the cycling, the dropped batteries are kept idle for three days at full charged condition and the OCVs are monitored. If the OCV drops more than or equal to 0.4*V* in three days, then those batteries are identified as faulty (ISC). Application of simple circuit theory on the battery electrical equivalent circuit (Fig. 2b) shows that the short circuit resistance for such cases will be less than 500*Ω*. If a battery does not develop ISC after the first drop, the same drop procedure is repeated until the battery becomes faulty or completely unusable.

### Random forest classifier

**Figure 7:**
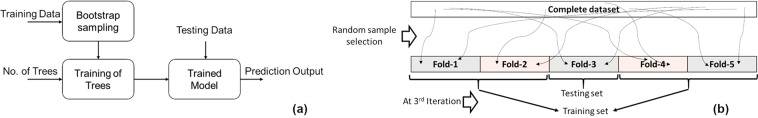
(**a**) Random Forest and (**b**) The five fold cross validation process.

The random forest (RF) algorithm was first introduced by Breiman in 2001^50^. RF uses a set of tree structures with small differences for the classification and regression purpose. Application of ensemble of trees makes the RF more robust and less prone to over fitting compared to a single tree. The flowchart in Fig. 7a illustrates the basic building blocks of the RF algorithm^50,63^.

The bootstrap sampling scheme used for RF implementation in this paper, divides the total training set of *N* samples into *N*_*t*_ groups of *N* samples. *N*_*t*_ is the number of trees present in the forest. Each group is formed by selecting samples randomly from the complete training set of *N* samples with replacements. Therefore, each group may contain multiple similar samples.

The number of trees (*N*_*t*_) in the forest and the number of features (*max*_*features*) used for splitting a node are the two most important parameters for the performance of the RF algorithm. Higher *N*_*t*_ gives better predictions at the cost of higher computational burden. *max*_*features* decides the randomness between the trees. Higher value of *max*_*features* gives nearly similar trees which may reduce robustness. Smaller value of *max*_*features* means deeper trees and increased computational burden. In the present implementation, the values of *N*_*t*_ and *max*_*features* are derived using the grid search among few suitable values.

To predict a class from the test data, first, all the trees make independent predictions with some probability values attached to each predictions. The class with highest overall probability is selected as the final prediction of the RF algorithm. Since the training of this algorithm is done offline, the computational complexity is determined by the testing stage of the RF algorithm on the smart phone and is given by *O*(*N*_*t*_. *D*) where D is the depth of the trees.

### Implementation and performance evaluation

The Python package scikit-learn is used for the RF implementation and performance evaluation. As discussed earlier, the performance of RF depends profoundly on the two important parameters, *N*_*t*_ and *max*_*features*. In the present implementation, *N*_*t*_ and *max*_*features* are derived by performing a grid search on the suitable range of values using the method *gridsearchCV* from the scikit-learn package. The performance for each set of parameters are evaluated by the five-fold cross validation (CVal), and the scores are evaluated as the AUC of the ROC curves. The values of *N*_*t*_ and *max*_*features* are derived using the healthy and ISC (with external resistance) data, and those values are used for the testing dataset of healthy and ISC (mechanically abused) battery data.

In the five-fold CVal scheme, the entire dataset is divided into 5 equal sets by randomly selecting samples from the full set without replacements. Training is performed on each combination of four sets and testing is performed on the remaining set. Therefore, the training and testing are performed total five times. CVal method reduces the chances of over training. A schematic diagram is shown in Fig. 7b to illustrate the CVal process.

RF assigns a probability value between 0 and 1 for each data sample classification. The probability value is compared with a threshold value (by default 0.5). If the probability value is more than the threshold, the data sample is classified as positive class otherwise as negative class. ROC is the false positive rate (FPR) 13 vs. true positive rate (TPR) 14 curve for different threshold values. AUC of ROC varies between 0 and 1. AUC closer to 1 means better classification of the samples.

13 $$FPR=\frac{FP}{FP+TN}$$

14 $$TPR=\frac{TP}{TP+FN}$$
